# MRI Manifestions Correlate with Survival of Glioblastoma Multiforme Patients

**DOI:** 10.3969/j.issn.2095-3941.2012.02.007

**Published:** 2012-06

**Authors:** Wen-bin Li, Kai Tang, Qian Chen, Shuai Li, Xiao-guang Qiu, Shao-wu Li, Tao Jiang

**Affiliations:** 1 Cancer Center, Beijing Shijitan Hospital, Capital Medical University, Beijing 100038, China; 2 Department of Neurosurgery, Beijing Tiantan Hospital, Capital Medical University, Beijing 100050, China; 3 College of Public Health, Peking University, Beijing 100871, China; 4 Department of Radiotherapy, Beijing Tiantan Hospital, Beijing 100050, China; 5 Department of Radiology, Beijing Tiantan Hospital, Beijing 100050, China

**Keywords:** glioblastoma multiforme, diffusion magnetic resonance imaging, survival

## Abstract

**Objective:**

To identify the correlation between magnetic resonance manifestation and survival of patients with glioblastoma multiforme (GBM).

**Methods:**

The magnetic resonance imaging (MRI) images of 30 glioblastoma patients were collected. Imaging features including degrees of contrasted area, edema surrounding the tumor, and intensity in T2-weighted imaging were selected to determine their correlation with patient survival. The relationship between imaging and survival time was studied using SPSS 19.0 software. Kaplan-Meier survival analysis and log-rank test were used to compare the survival curves.

**Results:**

Patients with ≤5% contrasted enhancement area of tumor had longer overall survival (OS) than those with >5% contrasted enhancement area of tumor. Patients without edema surrounding the tumor had longer OS than those with edema. Patients with tumor of hyperintensity and/or isointensity in T2-weighted imaging had longer OS than those with hyperintensity and/or isointensity and hypointensity.

**Conclusions:**

Some MR imaging features including degrees of contrasted area, edema surrounding the tumor, and intensity in T2-weighted imaging are correlated with the survival of patients with GBM. These features can serve as prognostic indicators for GBM patients.

## Introduction

Glioblastoma multiforme (GBM) is the most common primary brain tumor in adults, with an annual age adjusted incidence of 3.0 to 3.6 per 100,000 ^[^^1^^]^. GBM is classified by the World Health Organization as a grade IV glioma. Treatment modes include surgery, radiotherapy, and chemotherapy. The survival time of patients with GBM is significantly shorter than that of patients with lower-grade gliomas ^[^^2^^]^. Even within the same grade, the survival time for patients with GBM may also be highly variable. For more than 20 years, magnetic resonance imaging (MRI) has been used in the clinical diagnosis of gliomas to serve as a guide for clinical treatment. Most diagnoses for patients with malignant gliomas are followed by MRI. Thus, determining the relationship between survival and the appearance of tumor in MRI is of importance. Imaging features such as degrees of contrasted area, edema surrounding the tumor, and intensity in T2-weighted imaging can be easily collected from routine scans. This study aims to determine which imaging features are correlated with patient survival. This study was approved by the Ethics Committee of Beijing Tiantan Hospital, Capital Medical University.

## Patients and Methods

### Patients

Thirty patients diagnosed with GBM were selected from the Glioma Center, Beijing Tiantan Hospital, Capital Medical University, between January 2006 and July 2008. The ages of the patients ranged from 17 years to 70 years, with an average age of 45 years. All the patients had undergone operations in this center and were pathologically diagnosed with GBM WHO Grade IV. The diagnosis was confirmed by more than two neuropathologists. The patients also received radiotherapy and chemotherapy. During radiotherapy, each patient received 50 Gy to 60 Gy at 2 g per day, for 5.5-6.5 weeks. For chemotherapy, all the patients were administered with temozolomide at 75 mg per sqm of body every day during radiotherapy. For the next 6 months, they were administered with 150 mg to 200 mg of temozolomide per sqm of body per day at a rate of 5 days every month.

### Brain MRI

Brain MRI images of the 30 patients were obtained using a 1.5T scanner that in most cases included sagittal T1-weighted, axial T1-weighted, T2-weighted fast spin-echo, proton attenuation, and gadolinium diethylene triamine penta-acetic acid enhanced axial and coronal T1-weighted images. The scanner has a field of view of 24 cm and a matrix size of 256. All scans contained at least T1 pre- and post-contrast and T2-weighted images. MR imaging scans were read by 2 neuroradiologists who were blinded to patient outcome.

#### Image features

Five MRI image features such as degrees of contrasted area, edema surrounding the tumor, and intensity in T2-weighted imaging were selected for this study. The imaging definitions are as follows:

*i*) Degrees of contrasted enhancement area: When the contrasted enhancement area is ≤5% of the tumor, the image is “low-contrasted”; otherwise, it is “high/medium-contrasted.”

*ii*) Edema surrounding the tumor: When there is no evident edema surrounding the tumor, the image is defined as having “no edema”; otherwise, it has “edema.”

*iii*) Intensity in T2-weighted imaging: When the tumor shows hyperintensity or isointensity in T2-weighted imaging, the image is defined as “hyper or iso.” When the tumor shows hyperintensity or isointensity and hypointensity in T2- weighted imaging, the image is defined as hyper/iso/hypo.”

### Statistical analysis

The end point of the study was overall survival (OS), defined as the period from the date of surgery until the death from any cause or until September 2011. The statistical software SPSS 19.0 was used to estimate survival probabilities between the treatment options. To assess statistical significance of the effect of each feature on survival, the log-rank test was used for data analysis of patients who received surgery, radiotherapy, and chemotherapy. For all analyses, *P*<0.05 was considered as significant.

## Results

### The correlation between degree of contrasted enhancement area and patient survival

**Figure 1:**
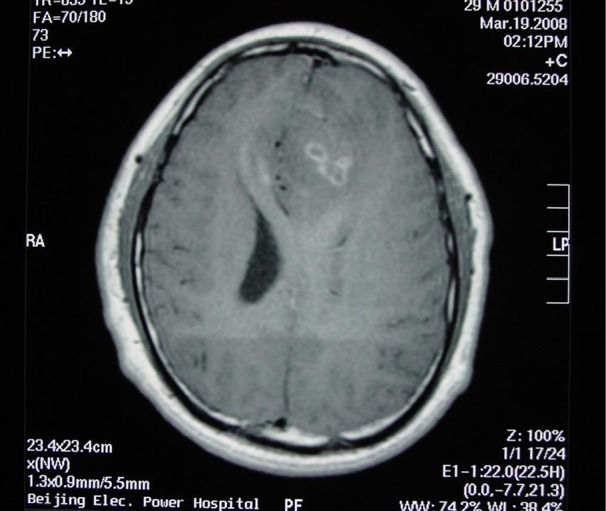
Contrasted enhancement ≤5%.

**Figure 2:**
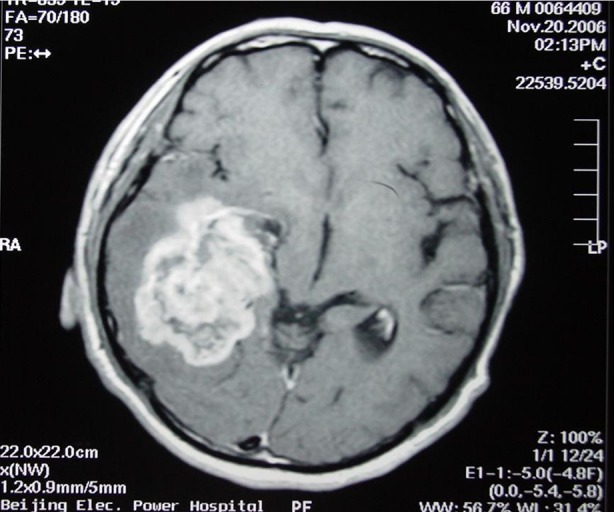
Contrasted enhancement >5%.

**Figure 3:**
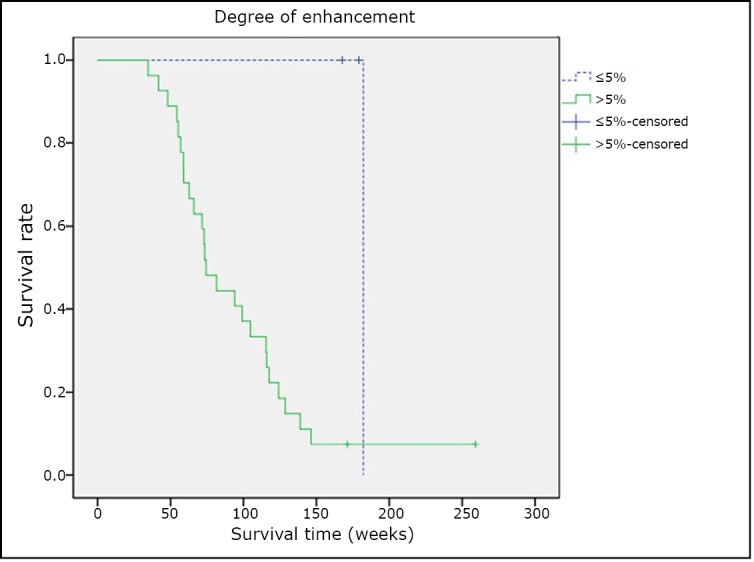
Tumor enhancement and patient survival.

The images of the tumor with ≤5% and >5% contrasted enhancement areas are shown in **Figures 1**** and ****2**, respectively. Of the 30 patients, the 3 with a tumor having ≤5% contrasted enhancement area had a medium survival time of 179.1 weeks; whereas the 27 with a tumor having >5% contrasted enhancement area had a medium survival time of 73.9 weeks. The two survival curves of these two groups were compared using log-rank teat (χ^2^=5.307, *P*=0.021). Based on the result, the difference in the OS between the two groups was statistically significant (**Figure 3**).

### The correlation between edema surrounding the tumor and patient survival

**Figure 4:**
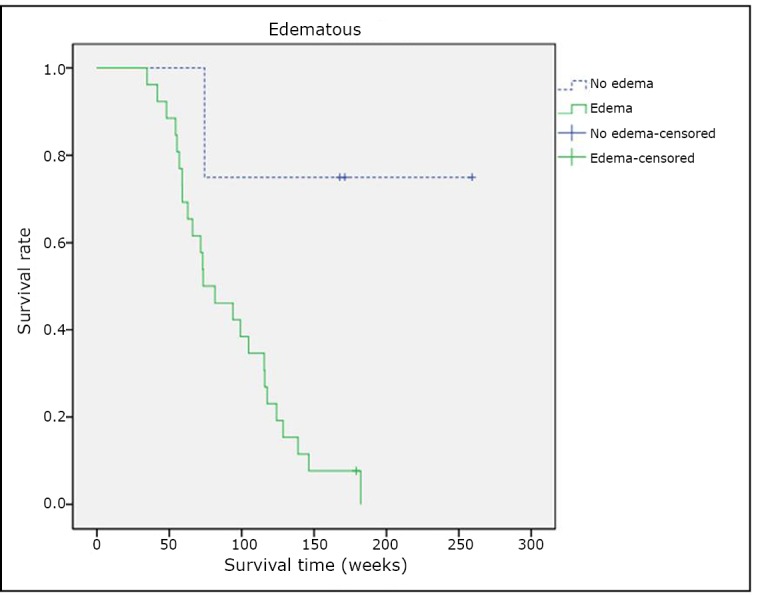
Edema surrounding the tumor and patient survival.

Among the 30 patients who underwent comprehensive treatment, “no edema” was found in 4 cases that had a medium survival time of 237.2 weeks; the other 26 cases were found to have “edema” and had a medium survival time of 120.9 weeks. The survival curves of these two groups were compared using log-rank test (χ^2^=6.617, *P*=0.013). The results also showed a statistically significant difference in the OS between patients with “no edema” and “edema” as shown in **Figure 4**.

### The correlation between intensity in T2-weighted imaging and patient survival

**Figure 5:**
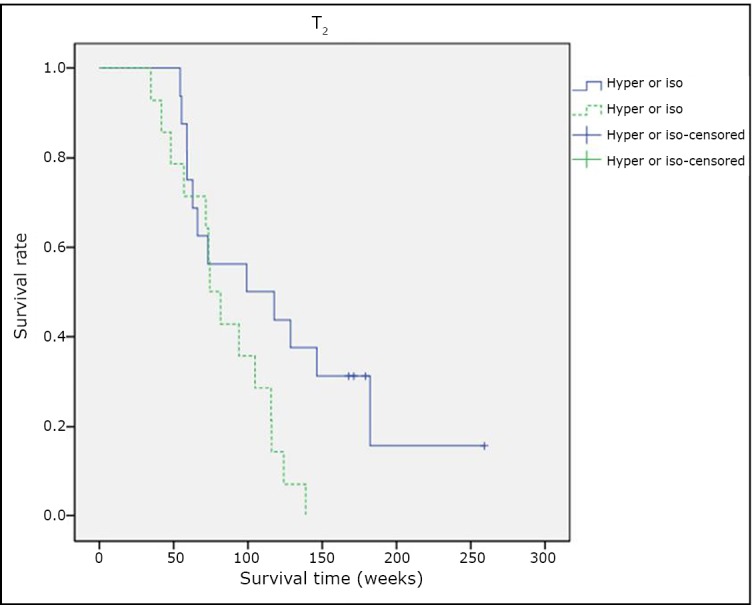
Intensity in T2 weighted imaging and patient survival.

Among the 30 patients undergoing comprehensive treatment, “hyper or iso” images in T2 were found in 16 cases, with a medium survival time of 108.4 weeks, while the other 14 cases were found to be “hyper/iso/hypo” with a medium survival time of 74.3 weeks. The survival curves of these two groups were compared using log-rank test (χ^2^=4.232, *P*=0.039). **Figure 5** shows a statistically significant difference in the OS of patients between “hyper or iso” and “hyper/iso/hypo.”

## Discussion

GBM is the most common primary malignant brain tumor in adults. Since 2005, the recommended treatment of GBM has been comprehensive therapy, which includes surgery followed by radiotherapy with concomitant and adjuvant chemotherapy ^[^^3^^]^. In a recent series of studies, it has been reported that within 2 years after primary surgery, most patients with GBM will experience tumor recurrence and eventually die of the tumor. The median OS rate is 9.9 months to 14.6 months ^[^^4^^–^^7^^]^. In this study, the OS of patients undergoing comprehensive treatment with surgery, radiotherapy, and chemotherapy is 92.8 weeks (21.7 months).

The prognostic factors for OS are reported to be patient age, functional status, grade of resection, type of treatment, and methylation status of the MGMT gene promoter ^[^^8^^–^^10^^]^. However, the survival benefit of tumor resection remains controversial^[^^11^^–^^13^^]^. MRI has been used for more than 20 years to reach a clinical diagnosis of brain glioblastoma and to guide clinical treatments of the tumor. It is the most commonly used means of non-invasive diagnostic imaging for the diagnosis of brain glioblastoma and may contain more information than image, particularly genetic information. In our previous study, some MRI features of brain glioblastoma were found to have a close relationship with GBM molecular pathology ^[^^14^^]^. The molecular pathology correlates with the recurrence and survival of GBM patients^[^^15^^]^.

This study tried to identify the relationship of MRI manifestations with survival of patients with GBM. Several imaging features were analyzed to determine which would be most useful as prognostic indicators. These selected features are easily determined from routine scans and would therefore be useful in everyday clinical practice. To better determine prognosis from routine imaging studies could make treatment decisions much easier to make. In an analysis of three imaging features of patients with GBM, it was found that the degrees of contrasted area, edema surrounding the tumor, and intensity in T2-weighted imaging were correlated with survival of patients with GBM. Diehn et al. ^[^^16^^]^ also demonstrated that patients with infiltrative radio phenotype have significantly worse survival times than their edematous counterparts. These MRI features could serve as prognostic indicators for GBM patients. Some appearances of tumors MRI are correlated with survival of GBM patients and could serve as prognostic indicators of GBM.
